# Supplementary material for: Momentum, volume and investor sentiment study for u.s. technology sector stocks—A hidden markov model based principal component analysis
Source: PLoS One. 2025 Sep 9;20(9):e0331658. doi: 10.1371/journal.pone.0331658 (PMC12419650; doi:10.1371/journal.pone.0331658)
Supplement: Attachment — Submitted filename: Response to Reviewers.docx [file pone.0331658.s002.docx]

Response to Reviewers

Dear editor and reviewers:

Thank you so much for your kind instructions about the potential revisions of my paper! We are very much appreciated! According to your valuable suggestions, we have made several changes to our paper based on the past version.

In our revision, we have included following citation and reference literature from 2020-2025 in our paper:

(1) Vidaurre D. A new model for simultaneous dimensionality reduction and time-varying functional connectivity estimation. PLOS Computational Biology. 2021; 17(4). <https://doi.org/10.1371/journal.pcbi.1008580>
(2) Kelly B, Moskowitz T, Pruitt S. Understanding momentum and reversal. Journal of Financial Economics, 2021; 140(3): 726-743. <https://doi.org/10.1016/j.jfineco.2020.06.024>
(3) Goyal A, Jegadeesh N, Subrahmanyam A. Empirical determinants of momentum: a perspective using international data. Review of Finance. 2025; 29(1): 241-273.

<https://doi.org/10.1093/rof/rfae038>

(4) Han, Y, Huang D, Huang D, Zhou G. Expected return, volume, and mispricing. Journal of Financial Economics. 2022; 143(3): 1295-1315. <https://doi.org/10.1016/j.jfineco.2021.05.014>
(5) Wang W, Su C, Duxbury D. Investor sentiment and stock returns: Global evidence. Journal of Empirical Finance. 2021; 63: 365-391. <https://doi.org/10.1016/j.jempfin.2021.07.010>
(6) Aggarwal D. Defining and measuring market sentiments: a review of the literature. Qualitative Research in Financial Markets. 2022; 14(2): 270-288. <https://doi.org/10.1108/QRFM-03-2018-0033>
(7) Seok S, Cho H, Ryu D. Dual effects of investor sentiment and uncertainty in financial markets. The Quarterly Review of Economics and Finance. 2024; 95:300-315. <https://doi.org/10.1016/j.qref.2024.04.006>
(8) Hultin H, Hult H, Proutiere A, Samama S, Tarighati A. A generative model of a limit order book using recurrent neural networks. Quantitative Finance. 2023; 23(6): 931-958. <https://doi.org/10.1080/14697688.2023.2205583>
(9) Lucchese L, Pakkanen M, Veraart A. The short-term predictability of returns in order book markets: A deep learning perspective. International Journal of Forecasting. 2024; 40(4): 1587-1621. <https://doi.org/10.1016/j.ijforecast.2024.02.001>
(10) Barber B, Huang X, Odean T, Schwarz C. Attention-Induced Trading and Returns: Evidence from Robinhood Users. The Journal of Finance. 2022; 77(6):3141-3190. <https://doi.org/10.1111/jofi.13183>
(11) Gao Z, Ren H, Zhang B. Googling Investor Sentiment around the World. Journal of Financial and Quantitative Analysis. 2020; 55(2): 549-580. <https://www.jstor.org/stable/26887960>
(12) Liu Q, Song H. Methods for aggregating investor sentiment from social media. Humanities and Social Sciences Communications. 2024; 11. https://doi.org/10.1057/s41599-024-03434-2

In this revision, we have made other changes as follows:

(1) We have reformatted the reference literature and made it coherent with PLOS One publication format. We have expanded our citation list based on the old version. Especially for the investor sentiment effect, we have added more literature in this part. These literature use either traditional reduce form model or PCA techniques, which are all classical literature in the behavior finance field.
(2) We have also included a paragraph to illustrate the contributions of our PCA-HMM study in the first part. We have summarized five aspects to represent the contributions of our research, in contrast with past investment sentiment papers, from the model setups, market regimes used, principal component selected, the consistent of data frequencies and risk management perspectives.
(3) We have reshaped the Fig 3 using software.
(4) We have also included Fig 4 in this revision, which represents the value of five principal components generated by our PCA-HMM to represent market sentiment. This could better help readers understanding the importance of including five components instead of the first principal component in investor sentiment calibration.
(5) We have included the risk measures, the risk aversion parameter, in our new version. This can assist us compare the market risk across different hidden states we defined and build relationships with volume and momentum factors.
(6) We have rerun all the graphs and tables due to yahoo finance database updated the “adjusted close price” into “close price” since our first submission in February, 2025.
(7) We also include the real-world implications of our PCA-HMM model in the very last part of our paper. We especially list the real-world implications from risk management, volume spike and investor sentiment aspects.

Thank you so much for your kind comments to the previous version again, which really helps us improve our paper! If you have any other revision suggestions, please also let us know! We would perfect this paper accordingly.

Thank you so much! Have a wonderful day!

Best regards,

Corresponding author
