# Supplementary material for: Momentum, volume and investor sentiment study for u.s. technology sector stocks—A hidden markov model based principal component analysis
Source: PLoS One. 2025 Sep 9;20(9):e0331658. doi: 10.1371/journal.pone.0331658 (PMC12419650; doi:10.1371/journal.pone.0331658)
Supplement: Attachment — Submitted filename: Response_07262025.docx [file pone.0331658.s003.docx]

Response to Editor and Reviewers

Dear editor and reviewers:

Thank you so much for your kind instructions about the potential revisions of my paper! We are very much appreciated! According to your valuable suggestions, we have made several improvements to our paper based on the foundation of past version.

At the very beginning, we would like to thank our editor to give us a precious opportunity to explain the importance of including certain reference literature in this new version. Regarding to the reference literature, we have made following improvements. Compared to the literature shown in previous version, we have included the reference literature from 2020-2025 as reviewers suggested, some important investor sentiment works in the investor sentiment section 2.3, in together with some works in discussion part 4.3.4 Discussions and real-world implications. The extra literature in the investor sentiment section 2.3 is very helpful for readers to obtain big picture to study investor sentiment using either index method or PCA method. The extra literature included in discussion part 4.3.4 draw connections between our findings with the findings in the past works.

The extra literature included in section 2.3 Investor sentiment compared to the past version are shown as follows:

(1) Tetlock P. Giving Content to Investor Sentiment: The Role of Media in the Stock Market. Journal of Finance. 2007; 62(3): 1139-1168. <https://doi.org/10.1111/j.1540-6261.2007.01232.x>
(2) Kurov A. Investor sentiment and the stock market' reaction to monetary policy. Journal of Banking & Finance. 2010; 34(1): 139-149. <https://doi.org/10.1016/j.jbankfin.2009.07.010>
(3) Da Z, Engelberg J, Gao P. The Sum of All FEARS Investor Sentiment and Asset Prices. The Review of Financial Studies. 2015; 28(1): 1-32.

<https://doi.org/10.1093/rfs/hhu072>

(4) Zhou G. Measuring Investor Sentiment. 2018; 10:239-259. <https://doi.org/10.1146/annurev-financial-110217-022725>
(5) Huang D, Jiang F, Tu J, Zhou G. Investor Sentiment Aligned: A Powerful Predictor of Stock Returns. 2015; 28(3): 791-837. <https://doi.org/10.1093/rfs/hhu080>
(6) Seok S, Cho H, Ryu D. Dual effects of investor sentiment and uncertainty in financial markets. The Quarterly Review of Economics and Finance. 2024; 95:300-315. <https://doi.org/10.1016/j.qref.2024.04.006>
(7) Lamont O, Stein, J. Investor Sentiment and Corporate Finance: Micro and Macro. The American Economic Review. 2006; 96(2): 147-151. <https://www.jstor.org/stable/30034631>
(8) Aggarwal D. Defining and measuring market sentiments: a review of the literature. Qualitative Research in Financial Markets. 2022; 14(2): 270-288. <https://doi.org/10.1108/QRFM-03-2018-0033>

The extra literature included in section 4.3.4 Discussions and real-world implications compared to the past version are shown as follows:

(1) Coudert V, Gex, M. Does risk aversion drive financial crises? Testing the predictive power of empirical indicators. Journal of Empirical Finance. 2008; 15(2): 167-184. <https://doi.org/10.1016/j.jempfin.2007.06.001>
(2) Hoffmann A, Post T, Pennings J. Individual investor perceptions and behavior during the financial crisis. Journal of Banking & Finance. 2013; 37(1): 60-74. <https://doi.org/10.1016/j.jbankfin.2012.08.007>
(3) Guiso L, Sapienza P, Zingales L. Time varying risk aversion. Journal of Financial Economics. 2018; 128(3): 403-421. <https://doi.org/10.1016/j.jfineco.2018.02.007>
(4) Madhavan A. Market microstructure: A survey. Journal of Financial Markets. 2000; 3(3):205-258. <https://doi.org/10.1016/S1386-4181(00)00007-0>
(5) Hultin H, Hult H, Proutiere A, Samama S, Tarighati A. A generative model of a limit order book using recurrent neural networks. Quantitative Finance. 2023; 23(6): 931-958. <https://doi.org/10.1080/14697688.2023.2205583>
(6) Lucchese L, Pakkanen M, Veraart A. The short-term predictability of returns in order book markets: A deep learning perspective. International Journal of Forecasting. 2024; 40(4): 1587-1621. <https://doi.org/10.1016/j.ijforecast.2024.02.001>
(7) Barber B, Huang X, Odean T, Schwarz C. Attention-Induced Trading and Returns: Evidence from Robinhood Users. The Journal of Finance. 2022; 77(6):3141-3190. <https://doi.org/10.1111/jofi.13183>
(8) Gao Z, Ren H, Zhang B. Googling Investor Sentiment around the World. Journal of Financial and Quantitative Analysis. 2020; 55(2): 549-580. <https://www.jstor.org/stable/26887960>
(9) Liu Q, Song H. Methods for aggregating investor sentiment from social media. Humanities and Social Sciences Communications. 2024; 11. https://doi.org/10.1057/s41599-024-03434-2

In addition, we specifically made following revisions according to the valuable suggestions from the reviewers:

1. With respected to the first question: Both citations and references should be reviewed to include recent literature from 2020-2025, we have cited several most recent works throughout the whole paper. In our revision, we have included following citation and reference literature from 2020-2025 in our paper:
(1) Vidaurre D. A new model for simultaneous dimensionality reduction and time-varying functional connectivity estimation. PLOS Computational Biology. 2021; 17(4). <https://doi.org/10.1371/journal.pcbi.1008580>
(2) Kelly B, Moskowitz T, Pruitt S. Understanding momentum and reversal. Journal of Financial Economics, 2021; 140(3): 726-743. <https://doi.org/10.1016/j.jfineco.2020.06.024>
(3) Goyal A, Jegadeesh N, Subrahmanyam A. Empirical determinants of momentum: a perspective using international data. Review of Finance. 2025; 29(1): 241-273.

<https://doi.org/10.1093/rof/rfae038>

(4) Han, Y, Huang D, Huang D, Zhou G. Expected return, volume, and mispricing. Journal of Financial Economics. 2022; 143(3): 1295-1315. <https://doi.org/10.1016/j.jfineco.2021.05.014>
(5) Wang W, Su C, Duxbury D. Investor sentiment and stock returns: Global evidence. Journal of Empirical Finance. 2021; 63: 365-391. <https://doi.org/10.1016/j.jempfin.2021.07.010>
(6) Aggarwal D. Defining and measuring market sentiments: a review of the literature. Qualitative Research in Financial Markets. 2022; 14(2): 270-288. <https://doi.org/10.1108/QRFM-03-2018-0033>
(7) Seok S, Cho H, Ryu D. Dual effects of investor sentiment and uncertainty in financial markets. The Quarterly Review of Economics and Finance. 2024; 95:300-315. <https://doi.org/10.1016/j.qref.2024.04.006>
(8) Hultin H, Hult H, Proutiere A, Samama S, Tarighati A. A generative model of a limit order book using recurrent neural networks. Quantitative Finance. 2023; 23(6): 931-958. <https://doi.org/10.1080/14697688.2023.2205583>
(9) Lucchese L, Pakkanen M, Veraart A. The short-term predictability of returns in order book markets: A deep learning perspective. International Journal of Forecasting. 2024; 40(4): 1587-1621. <https://doi.org/10.1016/j.ijforecast.2024.02.001>

(10) Barber B, Huang X, Odean T, Schwarz C. Attention-Induced Trading and Returns: Evidence from Robinhood Users. The Journal of Finance. 2022; 77(6):3141-3190. <https://doi.org/10.1111/jofi.13183>

(11) Gao Z, Ren H, Zhang B. Googling Investor Sentiment around the World. Journal of Financial and Quantitative Analysis. 2020; 55(2): 549-580. <https://www.jstor.org/stable/26887960>

(12) Liu Q, Song H. Methods for aggregating investor sentiment from social media. Humanities and Social Sciences Communications. 2024; 11. https://doi.org/10.1057/s41599-024-03434-2

2. With respected to the second question “The manuscript should be updated and improved. The discussion of findings should connect to real world applications and include partial implications”, we made following revisions. We have especially included the real-world implications of our PCA-HMM model in the very last part of our paper. We have listed the real-world implications in terms of risk management, volume spike and investor sentiment aspects. We illustrate the comparative advantages of our PCA-HMM in handling the market microstructure impact and dynamic sentiment shifts by drawing connections with unique characteristics of tech stocks properties. To illustrate the real-world implications in terms of risk management, we construct the absolute risk aversion parameters in our empirical application part and calculate its value under different states. Furthermore, we summarized the main contributions of our work in the first part of this paper made other improvements. The detail changes can be shown as follows:
(1) We have reformatted the reference literature and made it coherent with PLOS One publication format. We have expanded our citation list based on the old version. Especially for the investor sentiment effect, we have added more literature in this part. These literature use either traditional reduce form model or PCA techniques, which are all classical literature in the behavior finance field.
(2) We have also included a paragraph to illustrate the contributions of our PCA-HMM study in the first part. We have summarized five aspects to represent the contributions of our research, in contrast with past investment sentiment papers, from the model setups, market regimes used, principal component selected, the consistent of data frequencies and risk management perspectives.
(3) We have reshaped the Fig 3 using software.
(4) We have also included Fig 4 in this revision, which represents the value of five principal components generated by our PCA-HMM to represent market sentiment. This could better help readers understanding the importance of including five components instead of the first principal component in investor sentiment calibration.
(5) We have included the risk measures, the risk aversion parameter, in our new version. This can assist us compare the market risk across different hidden states we defined and build relationships with volume and momentum factors.
(6) We have rerun all the graphs and tables due to yahoo finance updated the “adjusted close price” into “close price” since our first submission in February, 2025.
(7) We also include the real-world implications of our PCA-HMM model in the very last part of our paper. We especially list the real-world implications from risk management, volume spike and investor sentiment aspects.

Thank you so much again for all your kind instructions to the previous version, which really helps us improve our paper! If you have any other revision suggestions, please also let us know! We would perfect our paper accordingly.

Thank you so much! Have a wonderful day!

Best regards,

Corresponding author
